# Development of a Monocyte Activation Test as an Alternative to the Rabbit Pyrogen Test for Mono- and Multi-Component *Shigella* GMMA-Based Vaccines

**DOI:** 10.3390/microorganisms9071375

**Published:** 2021-06-24

**Authors:** Danielle Carson, Sophie Myhill, Elena Palmieri, Francesca Necchi, Sjoerd Rijpkema, Francesca Micoli, Ida Karin Nordgren, Omar Rossi, Caroline Vipond

**Affiliations:** 1 The National Institute for Biological Standards and Control (NIBSC), South Mimms EN6 3QG, UK; sophie.myhill@nibsc.org (S.M.); Sjoerd.Rijpkema@nibsc.org (S.R.); Karin.Nordgren@nibsc.org (I.K.N.); 2 GSK Vaccines Institute for Global Health (GVGH) S.r.l., via Fiorentina 1, 53100 Siena, Italy; elena.x.palmieri@gsk.com (E.P.); francesca.x.necchi@gsk.com (F.N.); francesca.x.micoli@gsk.com (F.M.)

**Keywords:** outer membrane vesicle (OMV), generalised modules for membrane antigens (GMMA), monocyte activation test (MAT), reactogenicity, pyrogenicity

## Abstract

Generalised modules for membrane antigens (GMMA)-based vaccines comprise the outer membrane from genetically modified Gram-negative bacteria containing membrane proteins, phospholipids and lipopolysaccharides. Some lipoproteins and lipopolysaccharides are pyrogens; thus, GMMA-based vaccines are intrinsically pyrogenic. It is important to control the pyrogenic content of biological medicines, including vaccines, to prevent adverse reactions such as febrile responses. The rabbit pyrogen test (RPT) and bacterial endotoxin test (BET) are the most commonly employed safety assays used to detect pyrogens. However, both tests are tailored for detecting pyrogenic contaminants and have considerable limitations when measuring the pyrogen content of inherently pyrogenic products. We report the adaptation of the monocyte activation test (MAT) as an alternative to the RPT for monitoring the pyrogenicity of *Shigella* GMMA-based vaccines. The European Pharmacopoeia endorses three MAT methods (A–C). Of these, method C, the reference lot comparison test, was identified as the most suitable. This method was evaluated with different reference materials to ensure parallelism and consistency for a mono- and multi-component *Shigella* GMMA vaccine. We demonstrate the drug substance as a promising reference material for safety testing of the matched drug product. Our results support the implementation of MAT as an alternative to the RPT and use of the defined parameters can be extended to GMMA-based vaccines currently in development, aiding vaccine batch release.

## 1. Introduction

*Shigellae* spp. are the causative agents of shigellosis, characterised by stomach cramps, fever and diarrhoea, which can sometimes be bloody [1]. According to a study on the Global Burden of Diseases, Injuries and Risk Factors in 2016, shigellosis results in an estimated 212,438 deaths worldwide annually, 63,713 of which occur in children under five years of age [2]. Most *Shigella*-associated morbidity occurs in low- and middle-income countries (LMICs). An increasing prevalence of resistance to antibiotic treatments has led to *Shigella* being placed on the World Health Organisation (WHO) anti-microbial resistance priority list, highlighting a need for other options to control this disease [3]. There are currently no widely available licensed vaccines against *Shigella*, and as such, it has been prioritised by the WHO Product Development for Vaccine Advisory Committee (PDVAC) with the aim of focussing product development and abating policy and implementation gaps to accelerate vaccine availability [4].

In recent years, the GSK Vaccines Institute for Global Health (GVGH) has developed generalised modules for membrane antigens (GMMA)-based *Shigella* vaccines. GMMA particles are outer membrane vesicles (OMVs) produced by genetically modified Gram-negative bacteria [5,6,7]. An investigational *S. sonnei* GMMA vaccine (1790GAHB) was produced from attenuated *S. sonnei* strain 53G∆*virG*∆*tolR*∆*htrB*, which carries a deletion for the inner and outer membrane linkage protein TolR allowing enhanced blebbing of OMVs [8]. Shed GMMA particles have been detoxified by deletion of the late acyltransferase gene *htrB* in the parent strain resulting in penta- rather than hexa-acylated lipopolysaccharide [5,9]. Subsequent clinical trials have shown 1790GAHB to be well tolerated and immunogenic in adults from both Europe and Kenya, where shigellosis is endemic [10,11,12]. This success has led to the development of a four-component vaccine containing GMMAs from *S. sonnei* and three clinically relevant *S. flexneri* strains. 

By their nature, GMMA particles include several toll-like receptor agonists, most notably lipopolysaccharide, and thus, are inherently pyrogenic or fever-inducing [9]. In order to limit the risk of febrile reactions following administration, it is important to control the number of pyrogenic agents in parenteral medicine including vaccines. Three tests for assessing the pyrogenic content of medicinal products are described by the European Pharmacopoeia (Ph. Eur). Historically, the ‘gold standard’ has been the rabbit pyrogen test (RPT); other tests are: the bacterial endotoxins test (BET), also known as the Limulus amoebocyte lysate test (LAL), and the monocyte activation test (MAT) [13,14,15]. 

For the RPT, the product is administered intra-venously and the rabbit is monitored for increased body temperature, which provides a qualitative indication of pyrogenicity. The historic establishment of the RPT resulted in it being considered the “industry standard” and its use consumes around 400,000 rabbits worldwide annually [16]. Furthermore, the RPT was originally designed to detect contamination in products that should be pyrogen free by delivering parenterals intravenously to enhance pyrogenic sensitivity. However, vaccines are most commonly delivered via other administration routes, such as intramuscularly in the case of *Shigella* GMMA. Implementation of the RPT for the safety testing of the meningococcal OMV-based vaccine Bexsero gave a measure of batch consistency that is prone to false-positive results [17,18]. An adaptation to deliver a full human dose of vaccine intramuscularly to the rabbit (mRPT) was developed for testing 1790GAHB. Results demonstrated an acceptable average temperature rise of 0.5 °C in the first four hours post-immunisation, which subsequently diminished [8,19]. However, notable drawbacks to mRPT remain, including the use of animals and associated high biological variability, as well as species-dependent differences in TLR specificity, which may affect the translatability of results obtained in rabbits [20]. In recent years, increased scientific scrutiny surrounding the translatability and ethical implications of the RPT have led to increased uptake of the in vitro BET as a replacement.

The BET relies on a clotting reaction between factor C sourced from the *Limulus polyphemus* or *Tachypleus tridentatus* horseshoe crab in the presence of endotoxin. However, both species are known to be endangered. To overcome this ethical issue, the use of recombinant factor C (rFC, based on the gene sequence of the horseshoe crab) and a fluorimetric end-point detection method was accepted into the Ph. Eur in 2021 [21,22]. However, the BET and the rFC method are limited as a safety test due to their inability to detect non-endotoxin pyrogens. As both the RPT and BET were designed to detect pyrogenic contaminants in medicines, their use for inherently pyrogenic vaccines also requires extensive dilution of the product to an established non-pyrogenic dose.

The MAT was first introduced in the 1980s and included in the Ph. Eur. in 2010. This test quantifies in vitro a defined cytokine released by human monocytic cells following incubation with the test material [15,23,24]. Three MAT methods, A, B and C, are described: method A and B are quantitative and semi-quantitative tests, respectively, that utilise endotoxin as a comparative reference, whilst method C uses an established lot as a reference [15]. The MAT has benefits over the RPT and BET in that it employs the use of human cells and is, therefore, more physiologically relevant. It is also able to detect both endotoxin and non-endotoxin pyrogens and it can be implemented quantitatively. Furthermore, the use of the MAT falls in line with the ‘3Rs’ principle of Replacement, Reduction and Refinement [25]. In 2014, The National Institute for Biological Standards and Control (NIBSC) developed the MAT for batch release testing of Bexsero. However, successful implementation of the MAT as a replacement for the RPT requires optimisation and validation of the assay for the defined test product [15].

The Future Vaccine Manufacturing Research (FVMR) hub is a strategic partnership with researchers, industry and national centres, which works towards optimising vaccine manufacture and development to reduce the cost and increase accessibility of vaccines in LMICs. Through a FVMR hub-led collaboration between GVGH and NIBSC, we report the development of the MAT as a replacement for the RPT to be implemented in the consistency/safety testing of *Shigella* GMMA-based vaccines.

## 2. Materials and Methods

### 2.1. Preparation of GMMA Drug Substance 

GMMA drug substances (DS) were produced at 30 L scale from 2 mutants of *Shigella sonnei* 53G (called 1790 and 2929) and from mutants of three *Shigella flexneri* serotypes as previously described [26,27]. Purified GMMA were stored in saline (*S. flexneri* GMMA) or Tris buffer (*S. sonnei* GMMA) at −70 ± 10 °C. For accelerated stability studies, DS have been stored for 3 months at −20 °C, 4 °C or 37–40 °C until testing.

### 2.2. Formulation of the GMMA Drug Product

1790GAHB has been prepared as previously described [8]. Other GMMA have been formulated by adsorption in NaCl 154 mM NaH_2_PO_4_ 20 mM pH 6.5 on Alhydrogel at a final concentration of 0.7 mg/mL Al^3+^. Monovalent 1790GAHB formulations were prepared at 200 μg/mL total protein, corresponding to 12 μg/mL O-antigen concentration. Four-component drug product (DP) included *S. sonnei* 2929-GMMA and the three *S. flexneri* GMMA each at 30 μg/mL O-antigen. Four different *S. sonnei* 1790-GMMA fresh formulations were prepared: one 1790GAHB FF01, containing the same GMP GMMA DS lot (stored at −70 ± 10 °C) as used for 1790GAHB SH15-001 clinical lot (stored at 4 °C for >6 years), and three *S. sonnei* 1790-GMMA DP (DP batch A, B and C) using three different GMMA DS batches (all produced at 30 L scale, stored at −70 ± 10 °C). DP have been stored at 4 ± 2 °C or at 25 and 37–40 °C for up to three months in the accelerated stability studies performed (afterwards 4 ± 2 °C until testing).

### 2.3. Preparation of Peripheral Blood Mononuclear Cells

Blood samples were supplied in leukoreduction system chambers or apheresis cones from the Oxford blood transfusion centre (NHS Blood and Transplant, Oxford, UK) on the day of donation, and peripheral blood mononuclear cells (PBMC) were isolated on receipt at NIBSC. Full consent was given by the donors.

PBMC isolation was carried out as previously described [28]. Briefly, the bottom and top tube of the cone were cut, and blood was allowed to drip into 2 × 50 mL falcon tubes. Each 50 mL falcon tube was topped up to 45 mL with Heparin (5 IU/mL final concentration) in complete medium (RPMI supplemented with 100 U/mL penicillin, 100 μg/mL streptomycin, 10 mM HEPES, 1 × MEM non-essential amino acids (NEAA), 2 mM L-glutamine and 2% (*v*/*v*) human AB serum (Biowest, Nuaille, France)). The 90 mL total volume of blood-heparin medium solution was layered on 3 × 17 mL of Histopaque-1077 (Sigma Aldrich, Gillingham, UK) in 50 mL falcon tubes. PBMCs were isolated by density gradient centrifugation, washed three times with complete medium and resuspended in complete medium. Cells were quantified with Trypan blue, aliquoted in 50% human AB serum, 50% cryoprotectant (RPMI, L-glutamine, 2 mM L-glutamine, DMSO (16.28%)) to a density of 3 × 10^7^ cells/vial and stored in nitrogen vapour until use.

### 2.4. Monocyte Activation Test

PBMC aliquots were thawed as previously described into chilled thawing media (RPMI supplemented with 1% (*v*/*v*) human AB serum (Biowest, Nuaille, France) [29]. The suspension was centrifuged (240× *g*, 8 min, 10 °C with minimal breaking), supernatant was discarded and cell pellet was resuspended in 10 mL of complete cell culture media (RPMI supplemented with 100 U/mL penicillin, 100 μg/mL streptomycin, 10 mM HEPES, 1 × MEM NEAAs, 2 mM L-glutamine and 2% (*v*/*v*) human AB serum (Biowest, Nuaille, France)). An aliquot of cell suspension was counted using Trypan blue to measure cell number and viability. The remining cell suspension was then diluted to 1 × 10^6^ cells/mL in complete cell culture medium and 125 μL was added per well of 96-well round-bottomed polypropylene cell culture plates (Corning, Flintshire, UK). GMMA vaccine drug product (DP) and/or drug substance (DS) were gently mixed, diluted to an initial donor-dependent working concentration in complete cell culture medium and two-fold serially diluted in a pyrogen-free 96 deep-well plate (Greiner Bio-One, Gloucestershire, UK). Endotoxin dilutions were prepared from a 2000 IU/mL stock solution of endotoxin International Standard (IS, NIBSC 10/178), vortexed for 2 min, diluted to an initial working concentration of 5 IU/mL and two-fold serially diluted as described for the GMMA vaccine. A 1 mg/mL stock solution of Pam3CysSerLys4 (Pam3CSK4) (Invivogen, Toulouse, France) was diluted to an initial working concentration of 250 ng/mL in complete cell culture medium followed by 3-fold serial dilutions. Following preparation, 125 μL of the test sample was added to cell culture plates in quadruplicate. To determine the interference of the alum adjuvant, Alhydrogel was diluted 10-fold and tested in isolation or spiked with endotoxin at a final concentration of 0.1 IU/mL. Moreover, 125 μL of Endotoxin IS (final concentration of 0.5 IU/mL) and complete medium alone were also added to the cell culture plates in quadruplicate as a positive and negative control, respectively. Plates were incubated at 37 °C, 5% CO_2_ for 18–21 h in a humid atmosphere.

Following incubation, supernatants were removed from the cell culture plate and stored at −20 °C or analysed immediately for interleukin-6 (IL-6) content by sandwich enzyme linked immunosorbent assay (ELISA) as previously described [30]. Briefly, supernatants (diluted 1/10 in complete medium) were analysed alongside IL-6 IS (NIBSC, 89/548) dilutions from 62.5 to 4000 pg/mL using an in-house mouse monoclonal capture and sheep polyclonal (directly conjugated to horseradish peroxidase, HRP) detection antibody pair. Tetramethylbenzidine was used as the HRP substrate and the reaction stopped using 1 M H_2_SO_4_. The absorbance was measured at 450 nm (using 540 nm as a reference) on the Multiskan GO microplate reader (Thermo Fisher Scientific, Loughborough, UK).

### 2.5. Data Analysis

Outliers within the four replicates for each sample or reference dilution were excluded by Dixon’s Q test (α = 0.02). CombiStats v. 6.0 (European Directorate for the Quality of Medicines & HealthCare (EDQM), Council of Europe, Strasbourg, France) was used to determine endotoxin equivalents (EE, EU/μg protein) or relative pyrogenicity units (RPUs) and validity criteria. For RPU calculations, the reference standard was assigned an arbitrary value of 1 and a relative value was calculated for test samples. Sample results were considered valid if: regression; *p* < 0.05, non-linearity; *p* > 0.001, non-parallelism *p* > 0.001 relative to the reference batch. EE and RPUs were determined from IL-6 values for a minimum of six dilution points by four-parameter fit analysis.

## 3. Results

### 3.1. MAT Method A and B Cannot Be Used to Test the Shigella GMMA Vaccine

**Figure 1:**
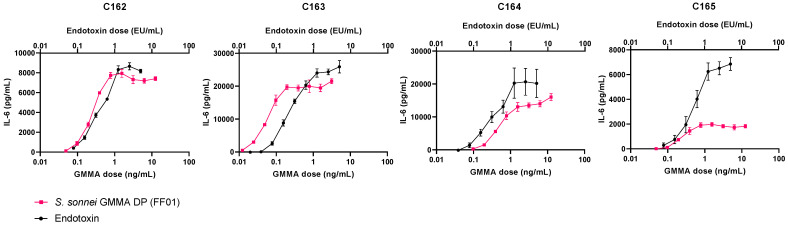
Dose response curves of peripheral blood mononuclear cells from four donors (C162-165) treated with *S. sonnei* GMMA drug product (DP, 1790GAHB FF01, pink squares) or endotoxin international standard (black circles). The highest concentration of *S. sonnei* GMMA DP was 12.5 ng/mL for donors C162, 164 and 165, and 3.125 ng/mL for donor C163.

To investigate the suitability of Ph. Eur. MAT method A or B, which both utilise endotoxin as a comparative reference, *S. sonnei* GMMA DP and DS were analysed using cryopreserved PBMCs from four independent donors and compared to the endotoxin IS. Individual rather than pooled donors were tested in order to capture donor variability for this novel vaccine. Given that the GMMA DP is formulated with Alhydrogel, we first confirmed that the adjuvant did not interfere with the release of IL-6 by PBMCs or its detection. For this purpose, endotoxin was spiked (at a final concentration of 0.1 IU/mL) into 10-fold serial dilutions of Alhydrogel. Results confirmed no effect of Alhydrogel on the release of IL-6 (Figure S1). The safety profile of the 1790GAHB SH15-001 clinical lot of *S. sonnei* GMMA DP has previously been established in adults [10,11,12]. As the 1790GAHB SH15-001 lot was >6 years old, *S. sonnei* GMMA DP was freshly formulated from the 1790-GMMA DS that was used to produce the clinical lot and has been stored at −80 °C. This lot was termed 1790GAHB FF01 (FF01) and tested utilising endotoxin as a reference. A nine-point two-fold dose response curve was defined for *S. sonnei* GMMA DP (FF01) with a range of 0.003 and 12.5 ng/mL total protein (Figure 1). The starting concentration for DP and DS was optimised for each donor due to the variable response to *Shigella* GMMA. For the endotoxin IS reference, a nine-point two-fold dose response curve ranging from 0.02 to 5 endotoxin units (IU/mL) was used for all donors (Figure 1). Results showed significant non-parallelism for two out of four donors (Table 1). In addition, inter-donor variability for endotoxin equivalents was high, with a geometric coefficient of variation (GCV) of 227.77%. Notably, inter-donor variability was independent of the endotoxin reference as it was also observed across the dose response curves for *S. sonnei* GMMA DP (FF01). Donor C163 was identified as a high IL-6 responder and required a further 6-fold dilution of the GMMA DP compared to the other three donors. Endotoxin stimulates IL-6 release via TLR4, whereas 1790GAHB GMMA have been shown to primarily activate TLR2 [9]. When TLR1/2 agonist, Pam3CysSerLys4 (Pam3CSK4), was added to the PBMCs of the four donors, IL-6 responses mirrored those observed with *S. sonnei* GMMA DP (FF01) (Figure S2a). However, analysis of *S. sonnei* GMMA DP and DS against Pam3CSK4 as a reference also revealed significant non-parallelism in two out of four donors, suggesting that the *S. sonnei* GMMA stimulates PBMCs via a combination of TLRs (Figure S2b, Table S1). Therefore, we concluded that methods A and B are unsuitable to analyse the reactivity of *Shigella* GMMA by MAT. 

### 3.2. The GMMA Drug Product Demonstrates a Decrease in IL-6 Release over Time

**Figure 2:**
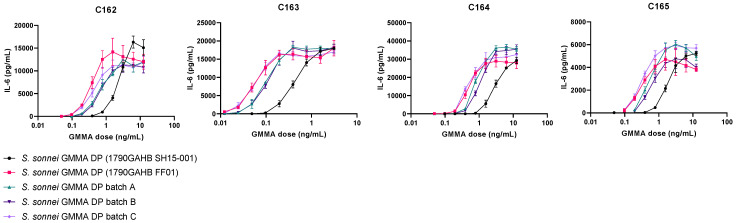
Dose response curves of peripheral blood mononuclear cells from four donors (C162-165) treated with the clinically tested *S. sonnei* GMMA drug product (DP, 1790GAHB SH15-001, black circles), *S. sonnei* GMMA DP (1790GAHB FF01, pink squares) or three independent GMMA DP batches (A: Green triangles, B: Dark purple inverted triangles, C: Light purple diamonds). The highest concentration of *S. sonnei* GMMA DPs was 12.5 ng/mL for donors C162, 164 and 165, and 3.125 ng/mL for donor C163.

Further experiments focused on implementing Ph. Eur. method C: the reference lot comparison test. Relative pyrogenicity was calculated from a minimum of six of the nine dilutions modelled by four-parameter fit analysis using Combistats version 6.0 (EDQM, Council of Europe). Therefore, we aimed to implement 1790GAHB FF01 as a reference standard. *S. sonnei* GMMA DP 1790GAHB FF01 was compared to 1790GAHB DP SH15-001 as well as three further *S. sonnei* 1790 GMMA DP batches (A, B and C), which were independently formulated from three separate batches of *S. sonnei* GMMA DS, to assess the batch-to-batch variability. DP batches A–C were all found to be within two-fold difference of *S. sonnei* GMMA DP (FF01) and had an inter-donor GCV of 32%, 18% and 35% for batch A, B and C, respectively (Figure 2, Table 2 and Table S2).

**Figure 3:**
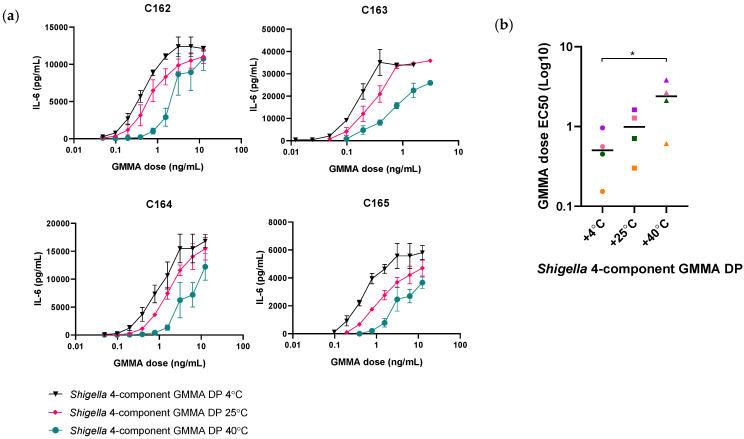
(**a**) Dose response curves of peripheral blood mononuclear cells from four representative donors, C162–165 (*n* = 10), treated with *Shigella* four-component GMMA drug product (DP) stored at 4 °C (black inverted triangles), 25 °C (pink diamonds) or 40 °C for 3 months. The highest concentration of *Shigella* four-component GMMA DP was 12.5 ng/mL for donors C162, 164 and 165, and 3.125 ng/mL for donor C163. A two-way ANOVA (*p* > 0.05) was carried out on OD values for statistical analysis. (**b**) Half maximal effective dose (EC50) values from the interleukin-6 response of four donors (each donor is represented by a different colour). A one-way ANOVA test was carried out (* *p* < 0.05).

Importantly, the 1790GAHB SH15-001 clinical lot was found to be 5.7 times less pyrogenic than the proposed reference, *S. sonnei* GMMA DP (FF01) (Table 2). As the DP is formulated with Alhydrogel, it is stored at 4 °C. At the time of testing, the 1790GAHB SH15-001 lot had, therefore, been stored at 4 °C for >6 years. Thus, the loss in IL-6 release is likely to be associated with changes to the DP over time. Indeed, testing of *Shigella* four-component GMMA DP samples subjected to accelerated degradation following exposure at 25 °C and 40 °C for 3 months did demonstrate a temperature-associated loss in IL-6 release of 1.4-fold and 5.2-fold, respectively, compared to the sample stored at 4 °C (Figure 3a, Table S3). Furthermore, GMMA stored at 40 °C showed a significant increase in the half maximal effective concentration (EC50) dose compared to GMMA stored at 4 °C (Figure 3b). The use of the DP as a reference batch would therefore require frequent monitoring and re-validation of fresh batches making a DP stored over a long period of time unsuitable as a reference standard.

### 3.3. IL-6 Response to Shigella Drug Substance Is Comparable to Drug Product and Can Be Proposed as a Reference for MAT Using Method C of Ph. Eur. Chapter 2.6.30

**Figure 4:**
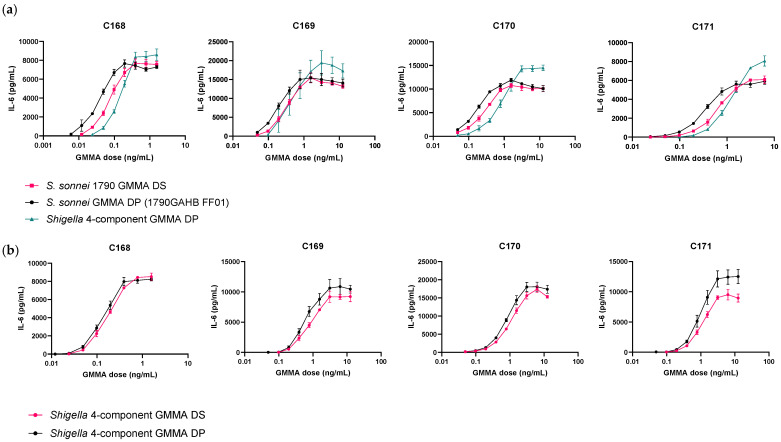
Dose response curves of peripheral blood mononuclear cells from four representative donors (*n* > 10); C 168-171 treated with *S. sonnei* GMMA (1790GAHB FF01) and *Shigella* four-component GMMA drug product (DP) and drug substance (DS). (**a**) Data show *S. sonnei* 1790 GMMA DS (pink sqaures), *S. sonnei* GMMA DP (1790GAHB FF01, black circles) and *Shigella* four-component GMMA DP (green triangles). The highest concentration of all *Shigella* GMMA vaccine products was 6.25 ng/mL for donors C169–171 and 1.56 ng/mL for donor C168. (**b**) Data show *Shigella* four-component DS (pink circles) and DP (black circles). The highest concentration of *Shigella* four-component GMMA DP and DS was 12.5 ng/mL for donors C169–171 and 1.56 ng/mL for donor C168.

As prolonged storage in Alhydrogel at 4 °C affects the ability of the DP to induce IL-6 release from human PBMCs, we next investigated the DS as an alternative reference, which is not formulated with Alhydrogel and can be stored at −80 °C. *S. sonnei* (1790GAHB FF01) and *Shigella* four-component GMMA DPs were tested using *S. sonnei* 1790 GMMA DS as a reference across PBMCs from 12 independent donors (Figure 4a and Table S4). On average, *S. sonnei* GMMA DP fell within a 0.61-fold range of the *S. sonnei* 1790 GMMA DS reference with an inter-donor GCV of 35.84% and an average RPU of 1.63. Comparatively, the *Shigella* four-component GMMA DP fell within a 1.34-fold range of the *S. sonnei* 1790 GMMA DS reference with a higher inter-donor GCV of 47.93% and an average RPU of 0.75. Of the 12 PBMC donors tested, parallelism between *S. sonnei* GMMA DP and the reference was observed in 100% of cases, whilst parallelism between the *Shigella* four-component GMMA DP and the reference was observed in only 25% of cases. These data indicate that *S. sonnei* 1790 GMMA DS is a suitable reference standard for *S. sonnei* GMMA vaccine DP only and is less suitable as a reference standard for multivalent *Shigella* GMMA vaccines.

We subsequently tested the *Shigella* four-component GMMA DS as a reference standard for *Shigella* four-component GMMA DP across PBMCs from 10 donors (Figure 4b, Table S5). On average, there was a 0.65-fold difference between DS and DP across donors with an inter-donor GCV of 33.92% and an average RPU of 1.54. Parallelism between DS and DP was maintained across 100% of donors, supporting the implementation of the ‘like-for-like’ DS as the reference for its corresponding DP.

### 3.4. Shigella GMMA Drug Substance Is More Stable Than the GMMA Drug Product

**Figure 5:**
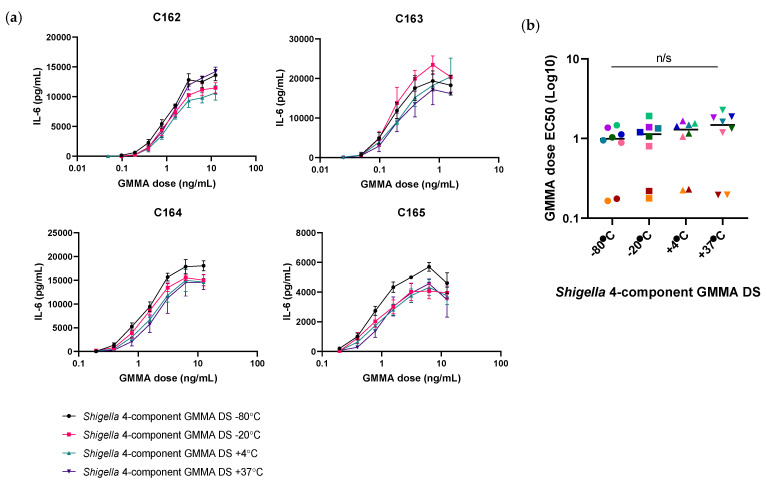
(**a**) Dose response curves of peripheral blood mononuclear cells from four representative donors (*n* = 8) treated with *Shigella* four-component GMMA DS stored at −80 °C (black circles), −20 °C (pink squares), 4 °C (green triangles) and 37 °C (purple inverted triangles). The highest concentration of *Shigella* four-component GMMA DP and DS was 12.5 ng/mL for donors C162, 164 and 165 and 1.56 ng/mL for donor C163. A two-way ANOVA (*p* > 0.05) was carried out on OD values for statistical analysis. (**b**) Half maximal effective dose (EC50) values from the interleukin-6 response of eight donors (each donor is represented by a different colour). A one-way ANOVA test was carried out (*p* > 0.05).

To investigate the stability of the DS, *Shigella* GMMA DS samples were stored at their recommended storage temperature of −80 °C or alternatively at −20 °C, 4 °C and 37 °C for three months and tested on PBMCs from eight donors. Following incubation, no significant difference was observed in the dose response curves of samples stored at −20 °C, 4 °C or 37 °C compared to −80 °C in each individual donor (Figure 5a, Table S6). Furthermore, no significant difference was observed in the GMMA dose EC50 values (Figure 5b). A 1.1-, 1.5- and 1.6-fold decrease in IL-6 release was observed at −20 °C, 4 °C and 37 °C, respectively, compared to −80 °C. These results demonstrate the increased stability of the *Shigella* GMMA DS over the DP.

## 4. Discussion

The GMMA-based vaccine platform is an attractive approach to address a public health need in LMICs due to its simplicity in manufacture and strong immunogenic potential [31]. Promising phase I and II trials of the monovalent *Shigella* vaccine have led to the development of a four-component *Shigella* vaccine which will provide coverage against the most prevalent *Shigella* serotypes [10,11,12]. For vaccine batch release, it is important to confirm that the pyrogenic response is maintained at safe levels. Historic use of the RPT for batch release of Bexsero, an OMV-based meningococcal B vaccine, proved problematic due to inconsistencies and false-positive test results contributed by extensive dilution of the vaccine product due to its intrinsic pyrogenic properties [17,32]. In addition, BET is not a suitable replacement for OMV-based vaccines due to the complexity of pathogen-associated molecular patterns (PAMPs) and the inability of this test to detect pyrogens of non-endotoxin origin. Therefore, there is a requirement to develop a MAT for the batch release of GMMA-based vaccines.

It is important to develop and validate MATs according to specific requirements to ensure suitability of the method and several variables must be defined, including an appropriate reference standard, dilution range and cytokine for detection. A panel of cytokines released by human PBMCs after stimulation with *S. sonnei* GMMA was previously analysed and high levels of IL-6, tumour necrosis factor-α, IL-1β and IL-8 were shown to be expressed [9]. In 2014, NIBSC established the IL-6-MAT for batch release of Bexsero. This methodology utilises non-heat-inactivated human serum, which has been shown to be optimal over the use of heat-inactivated serum, which shows a loss of reactivity towards endotoxins [33]. Furthermore, IL-6 was selected as a readout due to its full secretion into the surrounding media and because its presence is a measure of both endotoxin and non-endotoxin contaminants [32,34]. Building on experience of the Bexsero MAT and knowledge of GMMA-stimulated cytokines, IL-6 was implemented here as a suitable readout for GMMA-based vaccines.

Method C: the reference lot comparison test was demonstrated as the most appropriate methodology for the *Shigella* GMMA vaccines following the observed non-parallelism between endotoxin and the vaccine, likely due to differences in the activated pattern recognition receptors (PRR). Indeed, whilst endotoxin is a known TLR4 agonist, previous studies have confirmed the induction of a primarily TLR2 response by detoxified *Shigella* GMMA [9]. Interestingly, non-parallelism was also observed with the TLR2/TLR1 ligand Pam3CSK4. Thus, GMMA-stimulated IL-6 release is likely contributed by a combination of PAMPs, highlighting the complexity of immunostimulatory components present on the surface of the GMMA. Furthermore, method C is more suitable for situations of increased donor variability as observed here.

In pyrogenicity studies in rabbits, it has been found that DPs are less reactogenic than DS. For this reason, 1790GAHB was investigated as a potential reference batch. Indeed, 1790GAHB DP has been confirmed to have a good safety profile based on clinical trials performed so far in adults. However, it was deemed unsuitable as a reference in the MAT due to the observed reduction in IL-6 release over time, likely as a result of storage at 4 °C (due to formulation with alhydrogel). Studies have shown a reduction in rodent mortality when administered a mixture of endotoxin and aluminium hydroxide adjuvant compared to endotoxin alone [35,36]. It is possible that Alhydrogel masks GMMA by binding to exposed phosphate groups [36]. As Alhydrogel is only present in the DP, we used the 1790 GMMA DS as an alternative reference for *S. sonnei* GMMA DP. No interference of Alhydrogel with IL-6 release and detection was observed and similar RPUs were found for *S. sonnei* DS and DP. Whilst no significant difference was observed in the dose response curves of samples stored at increased temperatures compared to −80 °C, a 1.6-fold decrease in IL-6 release was observed for the DS stored at 37 °C, notably less than the 5.1-fold loss observed for the DP at 40 °C. Further investigations are being carried out to establish the full stability profile of the DS in real time; however, the results demonstrate the improved stability of the DS over the DP and show its promise as the comparative reference.

As such, we propose the *S. sonnei* DS as a suitable reference for *S. sonnei* DP vaccine batches. The *S. sonnei* DS was not suitable as a reference for the *Shigella* four-component vaccine, likely due to the difference in PAMP composition and subsequent PRR signalling. However, our data demonstrate the parallelism, linearity and comparability of RPUs between *Shigella* four-component GMMA DS and DP. Therefore, our data support the implementation of *Shigella* four-component DS reference batch as a reference for *Shigella* four-component DP vaccine batches. We hope that forthcoming clinical trials with the *Shigella* four-component GMMA vaccine will demonstrate its safety, as previously observed for 1790GAHB. Future work aims to validate and implement this methodology to ensure the safety of vaccine batches utilised in planned clinical trials. The use of this methodology could be further extended to accelerate the clinical testing of other GMMA-based vaccines, including nontyphoidal *Salmonella* spp.

## Figures and Tables

**Table 1:** Endotoxin equivalents (EE, IU/μg protein) and statistical analysis for individual donors in the monocyte activation test.

Donor	EE (IU/μg)	NP ^1^ (*p*-Value)	NL ^2^ (*p*-Value)	Equivalence Ratio with Standard	Correlation (*r*, Weighted)
C162	1419.4	0.000	0.000	1.291	0.949
C163	3137.6	0.263	0.050	0.914	0.956
C164	384.47	0.075	0.174	0.779	0.904
C165	235.57	0.000	0.000	−0.163	0.879

^1^ NP, non-parallelism; ^2^ NL, non-linearity.

**Table 2:** Monocyte activation test data showing the relationship of different *S. sonnei* GMMA drug products (DPs) with a proposed reference batch, 1790GAHB FF01.

GMMA Vaccine DP	Donor Average RPU ^1^	Fold Change	Inter-Donor GCV (%)
1790GAHB SH15-001	0.17	5.71	14.04
Batch A	0.65	1.54	31.94
Batch B	0.53	1.91	18.17
Batch C	1.02	0.98	35.07

^1^ *n* = 4.

## Data Availability

Not applicable.

## Supplementary Materials

The following are available online at https://www.mdpi.com/article/10.3390/microorganisms9071375/s1, Figure S1: PBMC response to Alhydrogel; Figure S2: PBMC response to TLR1/2 ligand Pam3CSK4; Table S1: RPU and statistical analysis for GMMA with Pam3CKS4 as a reference standard; Table S2: RPU and statistical analysis for different batches of S. sonnei GMMA DP with a reference S. sonnei DP batch as standard; Table S3: RPU and statistical analysis of accelerated degradation Shigella 4-component GMMA drug product samples; Table S4: RPU and statistical analysis of mono- and multi-component Shigella GMMA drug products with S. sonnei GMMA drug substance as a reference standard; Table S5: RPU and statistical analysis of Shigella 4-component GMMA drug product with Shigella 4-component GMMA drug substance as a reference standard; Table S6: RPU and statistical analysis of accelerated degradation Shigella 4-component GMMA drug substance samples.
