# Supplementary material for: Development of a Monocyte Activation Test as an Alternative to the Rabbit Pyrogen Test for Mono- and Multi-Component Shigella GMMA-Based Vaccines
Source: Microorganisms. 2021 Jun 24;9(7):1375. doi: 10.3390/microorganisms9071375 (PMC8306433; doi:10.3390/microorganisms9071375)
Supplement: Supplementary file 1 [file microorganisms-09-01375-s001.zip › microorganisms-1249993-supplementary.pdf]

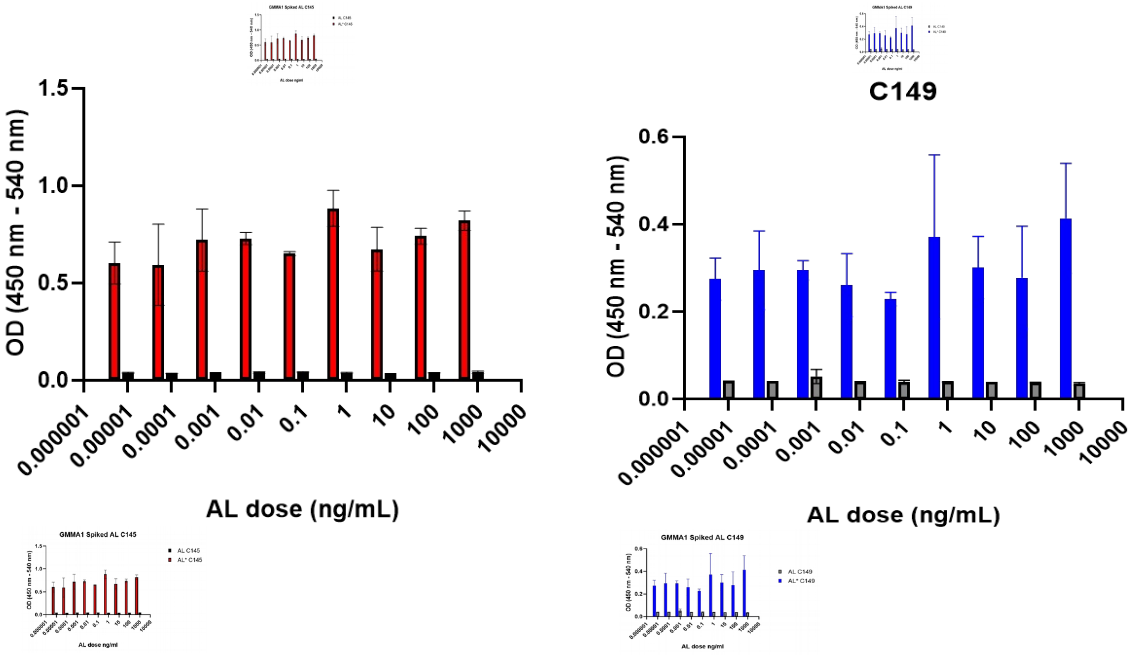

Figure S1. Response of PBMCs from two donors, C145 (left) and C149 (right) treated with 10-fold increasing doses of Alhydrogel only (grey bars) or Alhydrogel with spiked endotoxin (red and blue bars) at a final concentration of 0.1 IU/mL.

(a)

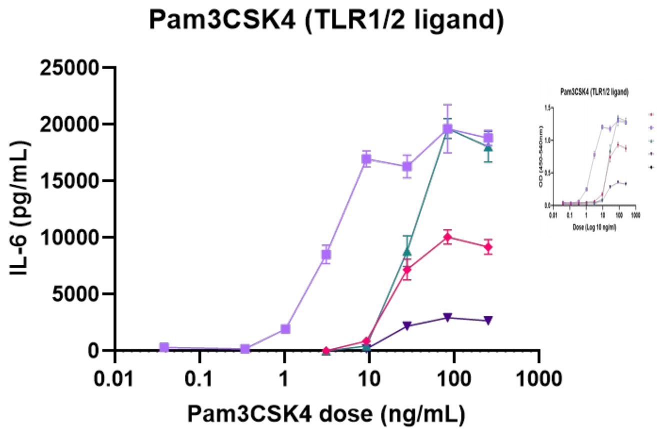

(b)

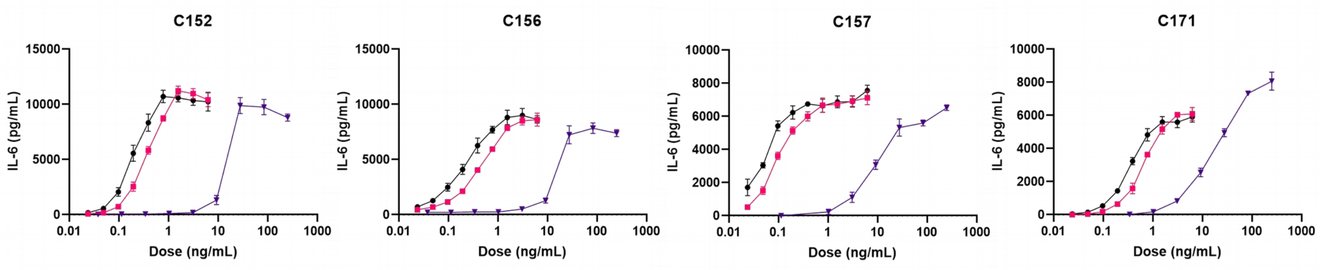

Figure S2. (a) Dose response curves of PBMCs from four donors (C162 - pink diamonds, C163 - light purple squares, C164 - green triangles, C165 - dark purple inverted triangles) treated with toll-like receptor (TLR)1/2 agonist Pam3CSK4. Nine three-fold dilutions of Pam3CSK4 between 0.04 and 250 ng/ml were tested. **(b)** Dose response curves of PBMCs from four representative donors; C152, 156, 157 and 171 treated with *S. sonnei* GMMA drug product (DP, 1790GAHB FF01, black circles), *S. sonnei* 1790 GMMA drug substance (DS, pink squares) and Pam3CSK4 (purple inverted triangles). The highest concentration of *S. sonnei* GMMA DP and DS was 6.25 ng/mL for all illustrated donors.

Table S1. MAT data showing relative pyrogenicity units (RPU) and statistical analysis for GMMA with Pam3CSK4 as a reference standard

IL-6 ANALYSIS			Donors			
			C152	C156	C157	C171
Pam3CSK4 AS STANDARD	1790GAHB FF01 DP	RPU	**8.03**	**15.71**	**22.66**	**52.71**
		Non-parallelism	0.128	0.000	0.016	0.000
		Non-linearity	0.962	0.402	0.000	0.036
		Eq. ratio with standard	0.845	0.430	0.828	1.779
		Correlation r (weighted)	0.934	0.856	0.921	0.914
	1790 GMMA DS	RPU	**4.58**	**8.45**	**10.45**	**25.66**
		Non-parallelism	0.866	0.000	0.002	0.000
		Non-linearity	0.057	0.012	0.000	0.029
		Eq. ratio with standard	1.016	0.462	0.813	1.686
		Correlation r (weighted)	0.943	0.867	0.932	0.942

Table S2. MAT data showing relative pyrogenicity units (RPU) and statistical analysis for different batches of *S. sonnei* GMMA drug product (DP) with a reference *S. sonnei* DP batch as standard

IL-6 ANALYSIS			Donors			
			C162	C163	C164	C165
1709GAHB FF01 AS STANDARD	1790GAHB SH15-001	**RPU**	**0.18**	**0.14**	**0.18**	**0.20**
		Non-parallelism (*p* value)	0.148	0.353	0.583	0.014
		Non-linearity (*p* value)	0.071	0.126	0.146	0.539
		Eq. ratio (standard = 1)	1.427	1.133	0.933	1.568
		Correlation *r* (weighted)	0.743	0.878	0.899	0.853
	BATCH A	**RPU**	**0.43**	**0.62**	**0.75**	**0.80**
		Non-parallelism (*p* value)	0.002	0.053	0.000	0.000
		Non-linearity (*p* value)	0.138	0.325	0.000	0.003
		Eq. ratio (standard = 1)	0.508	1.254	1.693	1.977
		Correlation *r* (weighted)	0.732	0.929	0.801	0.839
	BATCH B	**RPU**	**0.41**	**0.58**	**0.56**	**0.55**
		Non-parallelism (*p* value)	0.011	0.108	0.004	0.196
		Non-linearity (*p* value)	0.336	0.149	0.000	0.336
		Eq. ratio (standard = 1)	0.572	1.284	1.390	1.254
		Correlation *r* (weighted)	0.786	0.833	0.884	0.865
	BATCH C	**RPU**	**0.65**	**1.01**	**1.10**	**1.32**
		Non-parallelism (*p* value)	0.002	0.784	0.934	0.000
		Non-linearity (*p* value)	0.659	0.282	0.056	0.002
		Eq. ratio (standard = 1)	0.517	1.039	0.991	1.763
		Correlation *r* (weighted)	0.795	0.862	0.923	0.865

Table S3 MAT data showing relative pyrogenicity units (RPU) and statistical analysis of accelerated degradation *Shigella* 4-component GMMA drug product samples

IL-6 ANALYSIS			Donors									
			C162	C163	C164	C165	C168	C169	C170	C171	C152	C154
SHIGELLA 4-COMPONENT DP AS STANDARD	SHIGELLA 4-COMPONENT DP 25°C	RPU	**0.53**	**0.67**	**0.82**	**0.73**	**0.88**	**0.47**	**0.55**	**0.58**	**0.79**	**1.10**
		Non-parallelism (*p* value)	0.079	0.763	0.011	0.629	0.158	0.053	0.095	0.055	0.001	0.000
		Non-linearity (*p* value)	0.941	0.601	0.923	0.996	0.971	0.108	1.000	0.098	0.011	0.000
		Eq. ratio (standard = 1)	0.783	0.950	1.387	0.949	1.223	0.804	0.901	0.824	1.177	1.651
		Correlation *r* (weighted)	0.904	0.885	0.898	0.939	0.905	0.909	0.977	0.939	0.981	0.894
	SHIGELLA 4-COMPONENT DP 40°C	RPU	**0.13**	**0.20**	**0.20**	**0.19**	**0.42**	**0.09**	**0.17**	**0.12**	**0.12**	**0.30**
		Non-parallelism (*p* value)	0.799	0.000	0.098	0.706	0.090	0.012	0.343	0.024	0.100	0.050
		Non-linearity (*p* value)	1.000	0.509	0.952	0.996	0.997	0.103	1.000	1.000	0.277	0.251
		Eq. ratio (standard = 1)	0.963	0.604	1.313	0.952	1.184	0.829	0.952	0.829	1.130	0.871
		Correlation *r* (weighted)	0.906	0.906	0.861	0.932	0.952	0.969	0.988	0.970	0.964	0.972

Table S4 MAT data showing relative pyrogenicity units (RPU) and statistical analysis of mono– and multi–component *Shigella* GMMA drug products with *S. sonnei* GMMA drug substance as a reference standard

IL-6 ANALYSIS			Donors											
			C162	C163	C164	C165	C168	C169	C170	C171	C152	C154	C156	C157
1790 GMMA DS AS STANDARD	1790GAHB FF01	RPU	**1.51**	**0.66**	**1.34**	**1.42**	**1.95**	**1.69**	**1.77**	**1.74**	**1.81**	**1.62**	**2.15**	**1.96**
		Non-parallelism (*p* value)	0.138	0.148	0.880	0.982	0.456	0.889	0.217	0.054	0.526	0.745	0.506	0.472
		Non-linearity (*p* value)	0.198	0.065	0.059	0.032	0.565	0.353	0.252	0.569	0.042	0.003	0.840	0.256
		Eq. ratio (standard = 1)	1.138	0.881	0.989	0.998	0.943	1.017	1.064	0.857	0.962	1.036	0.959	1.060
		Correlation *r* (weighted)	0.950	0.939	0.955	0.940	0.966	0.903	0.986	0.957	0.973	0.924	0.971	0.949
	4-COMPONENT	RPU	**0.39**	**0.40**	**1.37**	**0.84**	**0.59**	**0.99**	**0.52**	**0.85**	**1.11**	**0.70**	**0.67**	**0.56**
		Non-parallelism (*p* value)	0.115	0.000	0.000	0.000	0.000	0.123	0.002	0.000	0.000	0.000	0.000	0.000
		Non-linearity (*p* value)	0.098	0.000	0.000	0.000	0.127	0.124	0.219	0.000	0.571	0.000	0.610	0.076
		Eq. ratio (standard = 1)	1.181	2.636	2.142	2.644	1.363	1.418	1.315	1.905	1.263	2.132	1.257	1.318
		Correlation *r* (weighted)	0.935	0.798	0.891	0.654	0.950	0.755	0.949	0.893	0.973	0.853	0.988	0.946

Table S5 MAT data showing relative pyrogenicity units (RPU) and statistical analysis of *Shigella* 4-component GMMA drug product with *Shigella* 4-component GMMA drug substance as standard

IL-6 ANALYSIS			Donors									
			C162	C163	C164	C165	C168	C169	C170	C171	C152	C154
SHIGELLA 4-COMPONENT DS AS STANDARD	SHIGELLA 4-COMPONENT DP	**RPU**	**2.09**	**1.27**	**1.64**	**2.52**	**1.18**	**1.54**	**1.44**	**1.65**	**1.08**	**0.98**
		**Non-parallelism (*p* value)**	0.009	0.300	0.057	0.002	0.074	0.010	0.056	0.001	0.020	0.964
		**Non-linearity (*p* value)**	0.112	0.815	0.678	0.994	0.092	0.716	1.000	0.943	0.339	0.128
		**Eq. ratio (standard = 1)**	0.909	1.239	1.349	1.450	0.909	1.321	1.124	1.406	1.180	1.003
		**Correlation *r* (weighted)**	0.867	0.866	0.875	0.919	0.980	0.933	0.977	0.939	0.962	0.977

Table S6 MAT data showing relative pyrogenicity units (RPU) and statistical analysis of accelerated degradation *Shigella* 4-component GMMA drug substance samples

IL-6 ANALYSIS			Donors							
			C162	C163	C164	C165	C168	C169	C170	C171
SHIGELLA 4-COMPONENT DS -80 AS STANDARD	SHIGELLA 4-COMPONENT DS -20°C	**RPU**	**0.73**	**1.15**	**0.78**	**0.56**	**0.70**	**1.58**	**0.68**	**0.90**
		Non-parallelism (*p* value)	0.062	0.062	0.026	0.000	0.039	0.029	0.800	0.150
		Non-linearity (*p* value)	0.042	0.169	0.121	0.194	0.106	0.837	1.000	0.989
		Eq. ratio (standard = 1)	0.856	1.510	0.847	0.572	0.659	1.889	0.959	1.162
		Correlation *r* (weighted)	0.958	0.793	0.967	0.917	0.793	0.747	0.854	0.946
	SHIGELLA 4-COMPONENT DS 4°C	**RPU**	**0.61**	**0.77**	**0.62**	**0.49**	**0.57**	**1.13**	**0.55**	**0.78**
		Non-parallelism (*p* value)	0.001	0.463	0.066	0.000	0.001	0.047	0.389	0.071
		Non-linearity (*p* value)	0.000	0.973	0.753	0.430	0.027	0.064	0.966	0.629
		Eq. ratio (standard = 1)	0.680	0.858	0.844	0.651	0.502	1.663	0.875	0.825
		Correlation *r* (weighted)	0.876	0.841	0.960	0.945	0.772	0.729	0.858	0.936
	SHIGELLA 4-COMPONENT DS 37°C	**RPU**	**0.82**	**0.69**	**0.53**	**0.53**	**0.79**	**0.41**	**0.60**	**0.60**
		Non-parallelism (*p* value)	0.185	0.136	0.053	0.034	0.014	0.029	0.337	0.586
		Non-linearity (*p* value)	0.180	0.924	0.330	0.083	0.868	0.750	1.000	0.990
		Eq. ratio (standard = 1)	1.122	0.700	0.755	0.703	0.731	0.680	1.168	1.074
		Correlation *r* (weighted)	0.949	0.797	0.885	0.863	0.911	0.852	0.860	0.926
